# Bacteriophages as Vehicles for Antibiotic Resistance Genes in the Environment

**DOI:** 10.1371/journal.ppat.1004219

**Published:** 2014-07-31

**Authors:** Jose Luis Balcazar

**Affiliations:** Catalan Institute for Water Research (ICRA), Scientific and Technological Park of the University of Girona, Girona, Spain; University of North Carolina at Chapel Hill School of Medicine, United States of America

## Occurrence and Impact of Antibiotic Resistance

Antibiotic therapy represents one of the most important medical advances of the 20th century and is a valuable resource in combating infectious diseases. Its therapeutic use, however, has been associated with the emergence of antibiotic-resistant bacteria. There are many naturally occurring ways in which susceptible bacteria become resistant to antibiotics, including by chromosomal mutations and/or horizontal gene transfer. The latter is largely, although not exclusively, responsible for the development of antibiotic-resistant bacteria through various processes such as conjugation, transformation, and transduction [1].

Transduction is a mechanism of genetic exchange, which is mediated by independently replicating bacterial viruses called bacteriophages, or phages [2]. Although the acquisition of antimicrobial resistance by transduction has been demonstrated in clinically relevant bacterial species, this mechanism in environmental settings has not been fully explored. However, cutting-edge genomic technologies such as high-throughput sequencing have recently led to significant advances in our understanding of the contribution of phages to the spread of antibiotic resistance genes (ARGs). This article will, therefore, describe the current knowledge on the emergence and spread of antibiotic resistance in the environment, with special emphasis on the role of phages in the mobilization of ARGs. Understanding sources and mechanisms of antibiotic resistance is critical for developing effective strategies for reducing their impact on public and environmental health.

## Genetic Exchange Mediated by Phages

Phages are viruses consisting of a DNA or RNA genome surrounded by a protein coat (capsid). They are the most abundant biological entities in the biosphere, with an estimated total population of 10^30^–10^32^ [3], [4]. Phages infect bacteria and either incorporate their viral genome into the host genome, replicating as part of the host (lysogenic cycle), or multiply inside the host cell before releasing new phage particles (lytic cycle).

**Figure 1:**
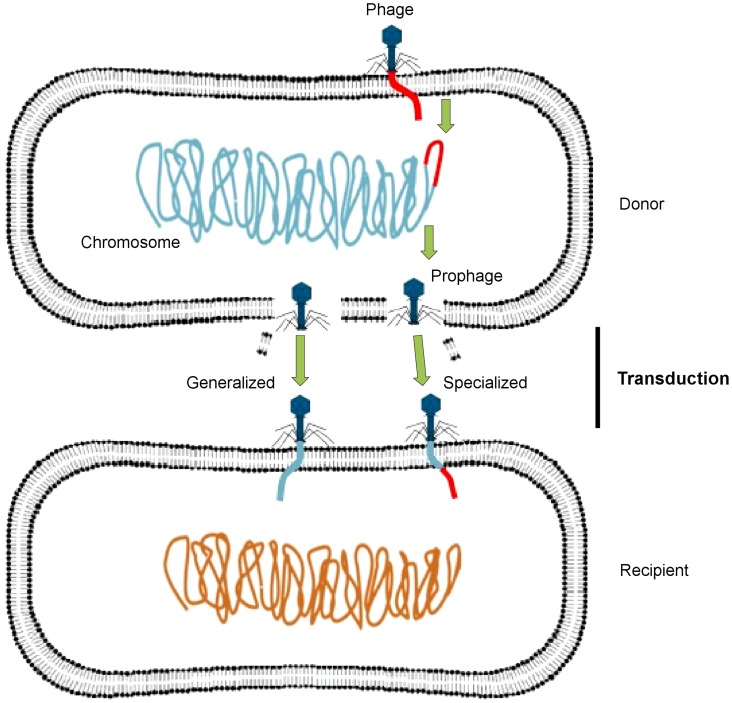
Transfer of DNA between bacteria via phages. A temperate phage inserts its genome (red) into the bacterial chromosome (blue-green) as a prophage, which replicates along with the bacterial chromosome, packaging host DNA alone (generalized transduction) or with its own DNA (specialized transduction). It then lyses the bacterial cell, releasing progeny phage particles into the surrounding environment. After lysis, these phages infect new bacterial cells, in which the acquired DNA recombines with the recipient cell chromosome (orange). This figure has been adapted from Frost et al. [2].

Phages may act as vectors for genetic exchange via generalized or specialized transduction, whereby a genetic trait is carried by phage particles from a donor bacterial cell to a recipient cell (Figure 1). Generalized transduction implicates the transfer of any portion of the donor genome to the recipient cell by either a lytic or lysogenic (temperate) phage, whereas specialized transduction involves only temperate phages, in which a few specific donor genes can be transferred to the recipient cell. A specialized transducing phage produces particles that carry both chromosomal DNA and phage DNA and contains only specific regions of the bacterial chromosome located adjacent to the prophage attachment site. Some temperate phages may also induce a change in the phenotype of the infected host, through a process known as lysogenic conversion [4].

Through these mechanisms, phages play an important role in the evolution and ecology of bacterial species, as they have the potential to transfer genetic material between bacteria. A metagenomic study has recently revealed that the viral metagenome (or virome) of antibiotic-treated mice was highly enriched for ARGs compared with that of nontreated control mice [5]. The authors also demonstrated that ex vivo infection of an aerobically cultured naive microbiota with phages from antibiotic-treated mice resulted in an increased bacterial resistance compared to infection with phages from the nontreated control. These findings clearly show that phages have significant implications for the emergence and spread of antibiotic resistance.

## Phages as Vehicles for Antibiotic Resistance Genes

Although antibiotic resistance is a natural phenomenon, the widespread use of antibiotics has contributed to the increase of antibiotic resistance in bacteria, including those causing infections in both humans and animals. Several studies suggest that antibiotic resistance found in clinical settings is intimately associated with the same mechanisms as those found in the environment [6]. In fact, the environment is continually exposed to a wide variety of antimicrobials and their metabolites through wastewater treatment plant (WWTP) discharges, agricultural runoff, and animal feeding operations, which may contribute to the emergence and spread of ARGs. Moreover, the large-scale mixing of environmental bacteria with exogenous bacteria from anthropogenic sources provides the ideal selective and ecological conditions for the emergence of resistant bacteria [7]. ARGs may be acquired and transferred among bacteria via mobile genetic elements (MGEs) such as conjugative plasmids, insertion sequences, integrons, transposons, and phages. Although the importance of these MGEs is widely recognized [1], the contribution of phages to the spread of ARGs has not been fully explored in environmental settings.

Recent findings, however, suggest that phages may play a more significant role in the emergence and spread of ARGs than previously expected. An extensive study using both sequence- and function-based metagenomic approaches revealed the presence of ARG-like genes in the virome of activated sludge, which could confer resistance to several antibiotics, including tetracycline, ampicillin, and bleomycin. None of the sequenced clones, however, conferred their predicted antibiotic resistance in *Escherichia coli*, likely due to incomplete cloning of the gene or lack of expression in *E. coli* [8]. Interestingly, a study using real-time PCR (qPCR) assays revealed the presence of two genes (*bla*_TEM_ and *bla*_CTX-M_) encoding β-lactamases and one gene (*mecA*) encoding a penicillin-binding protein in phage DNA from urban sewage and river water samples. In contrast to the previous study, the authors demonstrated that those ARGs (*bla*_TEM_ and *bla*_CTX-M_) from phage DNA were transferred to susceptible *E. coli* strains, which became resistant to ampicillin [9]. Another study of phage DNA from different hospital and urban treated effluents using qPCR assays showed the presence of high levels of genes (*bla*_TEM_, *bla*_CTX-M_ and *bla*_SHV_) conferring resistance to β-lactam antibiotics, as well as genes (*qnrA*, *qnrB* and *qnrS*) conferring reduced susceptibility to fluoroquinolones [10]. Likewise, a recent study demonstrated the presence of the *qnrA* and *qnrS* genes in phage DNA from fecally polluted waters and the influence of phage-inducing factors on the abundance of those ARGs. They observed that urban wastewater samples treated with chelating agents, such as EDTA and sodium citrate, showed a significant increase in the copy number of those ARGs in phage DNA compared to the nontreated samples [11]. Taken together, these studies not only suggest that anthropogenic inputs may facilitate the emergence of ARGs but also demonstrate the contribution of phages to the spread of ARGs into the environment.

## Metagenomic Exploration of Antibiotic Resistance in Environmental Settings

**Figure 2:**
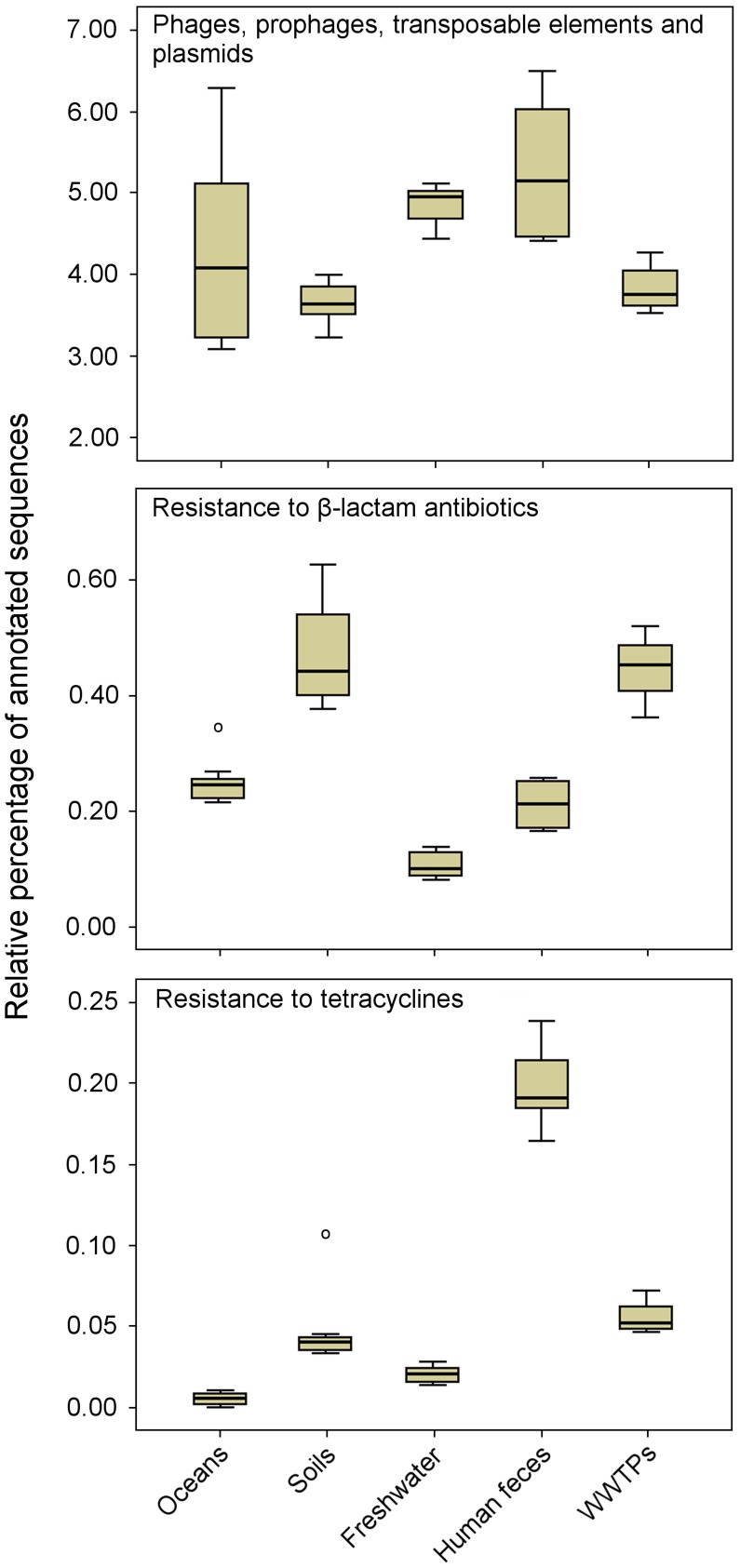
Metagenomic exploration of the resistome from human and environmental sources. Relative distribution of reads assigned to three functional subsystems among 27 metagenomes (based on MG-RAST annotation, *E*-value  =  10^−5^). Data are normalized by the total annotated sequences and are expressed as a percentage. The horizontal line in each box plot represents the mean of the relative distribution in each of the five environments (oceans, soils, freshwater, human feces, and WWTPs). The 27 metagenomes used for the analysis are available at http://metagenomics.anl.gov [13]. Accession numbers for oceans: 4441573.3, 4441574.3, 4441576.3, 4441577.3, 4441591.3, and 4443729.3; soils: 4441091.3, 4445990.3, 4445993.3, 4445994.3, 4445996.3, and 4446153.3; freshwater (rivers): 4511251.3, 4511252.3, 4511254.3, 4511255.3, 4511256.3, and 4511257.3; human feces: 4440595.4, 4440460.5, 4440611.3, 4440614.3, 4440825.3, and 4461119.3; and WWTPs: 4455295.3, 4463936.3, and 4467420.3.

Shotgun sequencing of metagenomic DNA offers significant advantages over culture-dependent methods, because it allows a better understanding of the structure and function of naturally occurring microbial communities [12]. This has resulted in the sequencing of thousands of metagenomes, which are now available via public platforms such as MG-RAST [13] and IMG/M [14]. These sequenced metagenomes can be used to explore the resistome in diverse environments. As an example, a comparison of 27 metagenomes (data available publically) corresponding to several projects and environments using the MG-RAST platform [13] revealed a relatively high proportion of sequences related to MGEs, including phages, among microbial communities from different natural environments (Figure 2). These results suggest that natural environments are a substantial source of MGEs, which may contribute to the horizontal transfer and spread of ARGs. The comparative analysis also revealed that the sequences related to genes conferring resistance to β-lactam antibiotics were detected more frequently among microbial communities from soil and WWTP environments than those from oceans, freshwaters, and human feces. Likewise, the sequences related to genes encoding tetracycline resistance were more abundant among microbial communities from human feces.

Metagenomic approaches offer valuable tools to explore the microbial community composition, ARGs, and MGEs in different environments. These environments may be further analyzed via clone libraries and functional screening in order to determine the role of MGEs (i.e., phages) as vehicles for ARGs. Therefore, high-throughput technologies, such as functional metagenomics, will provide unprecedented opportunities to elucidate the mechanisms and pathways by which antibiotic resistance evolves and spreads.

## Conclusions and Future Perspectives

It is now clearer than ever that the environment is a vast reservoir of resistant organisms and their associated genes. Moreover, there is increasing evidence that ARGs found in human microbial communities are likely to have been acquired from environmental sources [15], [16]. In fact, the use of metagenomic data generated via high-throughput sequencing has revealed that most ARGs found in nonpathogenic soil bacteria have perfect nucleotide identity to ARGs from several human pathogens, suggesting that recent horizontal gene transfer via MGEs has occurred between those organisms [17]. Metagenomic studies also demonstrate that phages, which may act as vehicles for ARGs, are widely distributed in nature. As a consequence, further studies should include the role of phages in developing effective strategies and mitigating the emergence and spread of antibiotic resistance, because this phenomenon is a significant and growing public health concern. A better understanding of the mechanisms and factors involved in phage induction will be crucial to reach these goals.
